# An Unusual Cause of Melena: Adjacent Sigmoid Tubular Adenomas With Moderate Dysplasia—A Case Report

**DOI:** 10.1002/ccr3.73138

**Published:** 2026-07-09

**Authors:** Ahmad Abbas, Mohammad Jaradat, Hidaya Kmail, Sameer Nairat, Razan Haj, Firas Jallad, Osama Qudaih, Jaser Amro, Baraa Diab, Mohanad Nazzal

**Affiliations:** ^1^ Fourth‐Year Medical Student, Faculty of Medicine Arab American University (AAUP) Jenin Palestine; ^2^ Gastroenterologist, Department of Internal Medicine Jenin Governmental Hospital Jenin Palestine; ^3^ Pathology Department Rafidia Surgical Hospital Nablus Palestine

**Keywords:** colonoscopy, lower gastrointestinal bleeding, melena, moderate dysplasia, sigmoid colon, tubular adenoma

## Abstract

Melena usually indicates upper gastrointestinal bleeding but may rarely result from slow bleeding in the distal colon. When upper endoscopy is negative, colonoscopy is essential. Although uncommon, bleeding colonic adenomas should be considered, as endoscopic polypectomy can provide both diagnosis and definitive treatment.

## 1 Introduction

Melena, defined as black, tarry stools resulting from the digestion of blood, is a classic sign of upper gastrointestinal hemorrhage, with approximately 90% of the cases originating proximal to the ligament of Treitz [1]. However, in about 10% of patients, melena may originate from the distal small bowel, right colon, or even the left colon when slow bleeding and prolonged transit time allow for hemoglobin breakdown [2]. While colonic lesions, including tubular adenomas, are commonly encountered during colonoscopy, they are usually incidental and rarely cause clinically significant bleeding manifesting as melena [3]. Early recognition of lower GI bleeding causes is essential, as delayed diagnosis may lead to severe iron‐deficiency anemia or recurrent hemorrhage. When esophageogastroduodenoscopy (EGD) fails to identify a bleeding source, colonoscopy becomes the important next step in evaluating lower GI causes of melena such as polyps, AVMs, diverticulosis, or neoplasia [4]. Although rare, isolated case reports have documented sigmoid colon polyps as an unusual cause of melena [5].

We present a rare case of melena in a young female caused by adjacent tubular adenomatous sigmoid polyps with moderate dysplasia, successfully treated with endoscopic polypectomy, resulting in complete resolution of melena.

## 2 Case History/Examination

### 2.1 Chief Complaint (CC): Melena for 3 Days

A 28‐year‐old female, previously in her usual state of good health, presented with a three‐day history of black, tarry stools. She reported that prior to this episode; she had been completely asymptomatic with no gastrointestinal complaints. She denied recent travel, changes in diet, or exposure to sick contacts. The patient was not taking any regular medications, including non‐steroidal anti‐inflammatory drugs, anticoagulants, or over‐the‐counter supplements. She also denied any history of peptic ulcer disease, gastritis, or prior episodes of gastrointestinal bleeding. Additionally, she reported no recent abdominal trauma, falls, or injuries that could potentially explain the bleeding. There was no family history of colorectal cancer, inflammatory bowel disease, or hereditary polyposis syndromes.

She described the onset of melena as gradual, noting progressive darkening of her stools without associated abdominal pain or changes in bowel habits. She denied hematemesis, nausea, vomiting, unintentional weight loss, fever, or fatigue preceding the presentation. She did not smoke, consume alcohol, or use recreational drugs.

On admission, her vital signs were stable; she was hemodynamically stable with no orthostatic changes. Physical examination was notable only for mild conjunctival pallor, but no icterus, petechiae, or stigmata of chronic liver disease. Abdominal examination was unremarkable, with a soft, non‐tender abdomen and no palpable masses.

Initial laboratory investigations revealed normocytic anemia, with a hemoglobin level of 10.1 g/dL, hematocrit of 30.1%, and an MCV of 84 fL. Platelet count and white blood cell count were within normal limits. Coagulation profile including prothrombin time (PT) and international normalized ratio (INR), renal function tests, and liver function tests were within normal limits. A fecal occult blood test returned positive.

**FIGURE 1:**
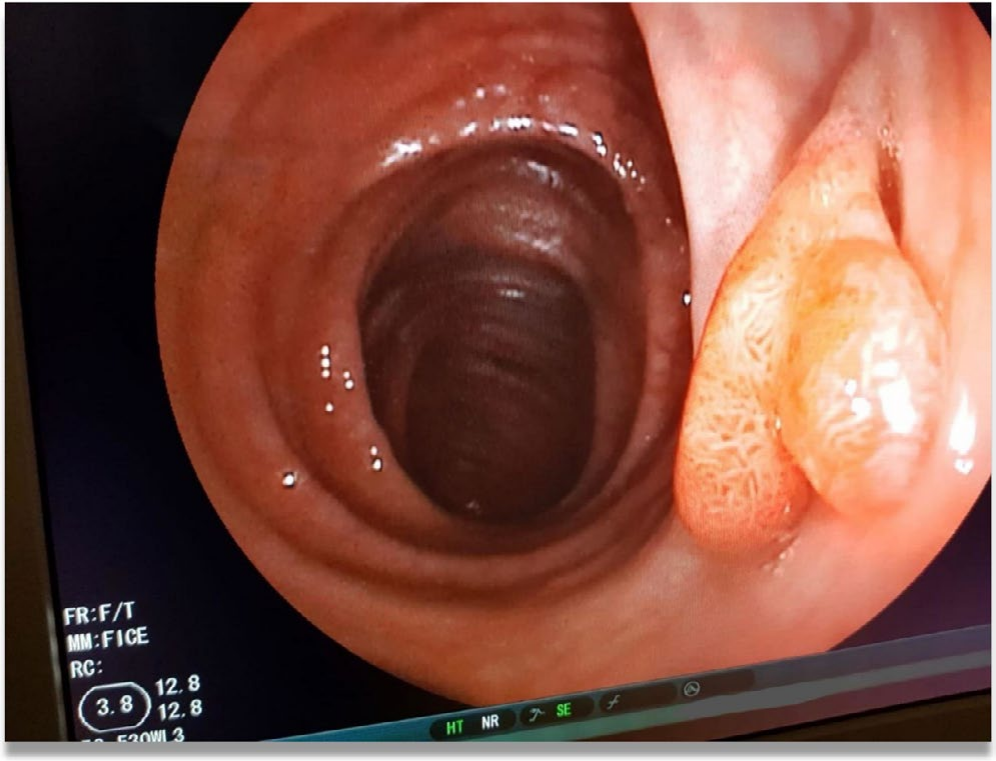
Colonoscopy image showing two adjacent sigmoid tubular adenomas.

Upper gastrointestinal endoscopy was performed to rule out an upper GI source and was entirely normal, with no evidence of bleeding, ulceration, or varices. Given the persistent melena and negative EGD, a colonoscopy was performed. In the sigmoid colon, two adjacent polyps were identified: one pedunculated polyp measuring approximately 2 cm in diameter, and one sessile polyp approximately 1 cm in diameter (Figure 1).

Both polyps were resected completely using hot snare polypectomy, the procedure was uneventful with immediate hemostasis achieved. Rest of colonoscopy up to terminal ileum was completely free with no additional lesions or sources of bleeding identified.

**FIGURE 2:**
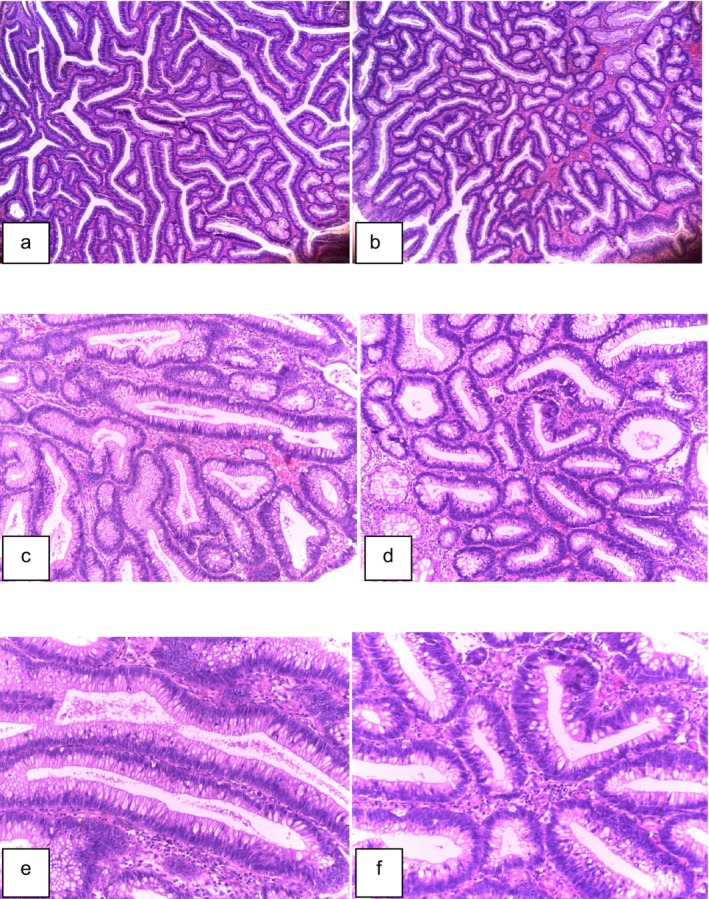
Photomicrograph of colonic polyps showing dysplastic epithelium (H&E; a, b ×4) composed of hyperchromatic and elongated nuclei (H&E; c, d ×10) arranged in pseudostratified manner (H&E; e ×20), with variable amounts of mucin loss (H&E; f ×20).

**FIGURE 3:**
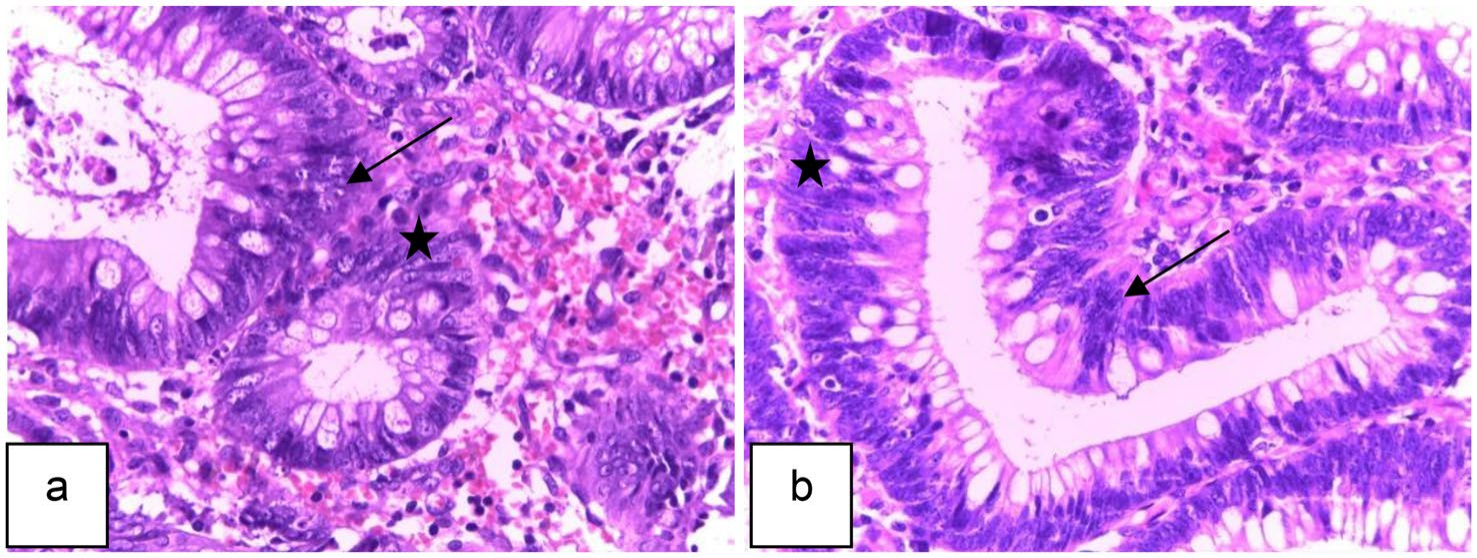
Photomicrograph showing cytologic changes of the dysplastic epithelium including vesicular chromatin (arrow) (H&E; a ×40), loss of basal polarity (star) (H&E; a, b ×40) and prominent nucleoli in some cells (arrow) (H&E; b ×40) displayed a degree of moderate dysplasia.

**FIGURE 4:**
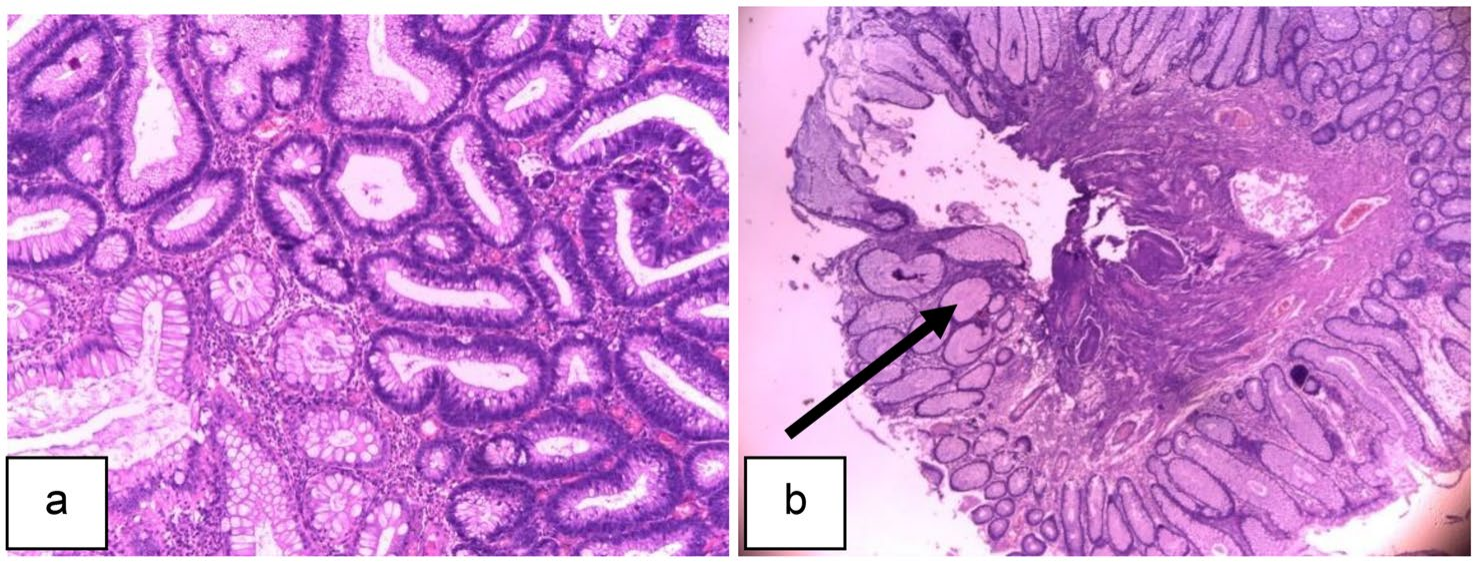
Photomicrograph showing an abrupt transition from normal colonic mucosa (left side) to dysplastic mucosa of the polyps (right side) (H&E; a ×4), and the base of polyps reveal normal mucosa and is free of dysplastic cells (arrow) (H&E; b ×4).

Gross examination revealed two polypoid lesions submitted for histopathologic evaluation. The resected lesions were polypoidal in nature, soft and friable in consistency, and dark red to tan in color. Due to fragmentation during resection and specimen processing, the pedunculated lesion was received in two pieces measuring 2 × 1 × 1 cm and 1.5 × 1 × 1 cm, while the sessile polyp measured 1 × 1 × 1 cm. Histopathologic examination showed a colonic mucosa covered with dysplastic epithelium (Figure 2a,b) composed of hyperchromatic and elongated nuclei (Figure 2c,d) arranged in a pseudostratified manner (Figure 2e), with variable amounts of mucin loss (Figure 2f). The cytologic changes of dysplastic epithelium including vesicular chromatin (Figure 3a), loss of basal polarity (Figure 3a,b), and prominent nucleoli in some cells (Figure 3b) displayed a degree of moderate dysplasia. An abrupt transition from normal colonic mucosa to dysplastic mucosa was identified (Figure 4a). The base of polyps was free of dysplastic glands (Figure 4b).

## 3 Differential Diagnosis

The differential diagnosis for melena in this patient included upper gastrointestinal bleeding due to peptic disease, erosive gastritis, or esophageal varices, as well as small bowel bleeding. Lower gastrointestinal causes such as colonic neoplasia, angiodysplasia, and inflammatory bowel disease were also considered. A normal upper endoscopy narrowed the bleeding source to the lower gastrointestinal tract.

## 4 Conclusion and Results (Outcome and Follow‐Up)

The patient's melena resolved completely within 24 h of the polypectomy. She was discharged with oral iron supplementation (ferrous sulfate 325 mg daily). At a 4‐week follow‐up visit, she remained asymptomatic with no recurrence of melena. A repeat complete blood count demonstrated significant improvement: Hemoglobin had risen to 12.3 g/dL, hematocrit to 37.5%, with all cell indices within normal ranges (Table 1), confirming resolution of the anemia.

**TABLE 1:** Complete blood count (CBC) on presentation and follow‐up.

Parameter	Reference range	Initial value	Follow‐up value
Hemoglobin (g/dL)	12.0–16.0 (female)	10.1	12.3
Hematocrit (%)	36–46	30.1	37.5
RBC Count (×10^12^/L)	4.1–5.5	3.57	4.37
MCV (fL)	80–100	84.3	85.8
MCH (pg)	27–33	28	28.1
MCHC (g/dL)	32–36	33.2	32.8
RDW (%)	11.5–14.5	12.2	13.3
WBC Count (×10^9^/L)	4.0–11.0	10.2	7.5
Neutrophils (%)	40–75	71.3	50.1
Lymphocytes (%)	20–45	26.3	39.6
Platelet Count (×10^9^/L)	150–400	363	324

This case highlights a rare cause of melena originating from sigmoid tubular adenomas with moderate dysplasia. Complete colonoscopic evaluation following a negative upper endoscopy is crucial, and endoscopic polypectomy provides definitive diagnosis and treatment.

## 5 Discussion

This case illustrates an unusual presentation of melena—bleeding from adjacent tubular adenomas in the sigmoid colon. While melena is a hallmark of upper GI bleeding, our case aligns with the literature describing the extremely rare cases stemming from sources distal to the ligament of Treitz like the left colon [1, 2]. Although melena is most commonly associated with upper gastrointestinal bleeding, distal colonic sources may occasionally produce melena when bleeding occurs slowly and intestinal transit time is prolonged. Under these circumstances, bacterial degradation and enzymatic oxidation of hemoglobin into hematin can occur before stool evacuation, producing the characteristic black tarry appearance. Such presentations are uncommon but have been described in cases of right‐sided colonic tumors, vascular malformations, and rarely bleeding adenomatous polyps [6].

Tubular adenomas are among the most common colonic polyps but are infrequently implicated in overt bleeding severe enough to cause melena [3, 7]. When they do bleed, it is often occult. The presence of moderate or high‐grade dysplasia is widely considered a marker of advanced adenoma (i.e., higher risk lesion), prompting more frequent surveillance, even though current guidelines do not explicitly link dysplasia grade to hemorrhage risk [8]. The definitive management in this case, hot snare polypectomy, served both diagnostic and therapeutic purposes, leading to immediate and sustained resolution of symptoms. This underscores colonoscopy's dual role as the gold standard for diagnosing unexplained melena after a negative EGD and as a primary therapeutic modality [4].

Although no active bleeding or clear stigmata of recent hemorrhage were visualized during colonoscopy, several findings support the adenomas as the most likely source of bleeding in this patient. Upper gastrointestinal endoscopy was completely normal, and colonoscopy did not reveal any additional lesions that could account for the melena. Furthermore, the patient's symptoms resolved promptly following endoscopic polypectomy, accompanied by a progressive normalization of hemoglobin levels during follow‐up. While this temporal association strongly suggests a causal relationship, the absence of direct endoscopic evidence of bleeding should be acknowledged, and therefore the conclusion remains based on the most plausible clinical correlation rather than definitive visual confirmation.

The resolution of melena and normalization of hemoglobin from 10.1 to 12.3 g/dL within 4 weeks post‐polypectomy provides strong objective evidence supporting the polyps as the definitive source. The patient's young age and absence of traditional risk factors make this presentation unusual, highlighting the importance of a complete GI workup regardless of age when clinical symptoms are evident. Furthermore, the finding of adjacent adenomas raises consideration of a field effect in the colonic mucosa, warranting adherence to established surveillance guidelines [9].

### 5.1 Limitations

One important limitation of this case is the absence of definitive endoscopic visualization of active bleeding from the polyp prior to resection. In addition, only a single colonoscopic image was available, and no additional pre‐resection endoscopic images demonstrating stigmata of recent bleeding were obtained. While no alternative bleeding source was identified during complete upper and lower endoscopic evaluation, the causal relationship between the adenomas and melena is therefore inferred primarily from the clinical course. The resolution of melena after polypectomy and normalization of hemoglobin levels strongly supports this association; however, intermittent bleeding from an undetected source cannot be completely excluded. Future reports with clearer endoscopic documentation of bleeding lesions would further strengthen this association.

## Author Contributions

**Ahmad Abbas:** writing – original draft. **Mohammad Jaradat:** writing – review and editing. **Hidaya Kmail:** writing – review and editing. **Sameer Nairat:** supervision. **Razan Haj:** formal analysis. **Firas Jallad:** data curation. **Osama Qudaih:** data curation. **Jaser Amro:** resources. **Baraa Diab:** data curation. **Mohanad Nazzal:** validation.

## Funding

The authors have nothing to report.

## Ethics Statement

This study is a single‐patient case report and did not require institutional ethical approval. This study conforms to the CARE guidelines.

## Consent

Written informed consent was obtained from the patient for publication of this case report and any accompanying images. A copy of the written consent is available for review by the Editor‐in‐Chief of this journal.

## Conflicts of Interest

The authors declare no conflicts of interest.

## Data Availability

All data generated analyzed during this study are included in this published article. Additional data, such as laboratory values and the patient consent form, are available from the corresponding author upon reasonable request.
